# Positional Assembly of Enzymes on Bacterial Outer Membrane Vesicles for Cascade Reactions

**DOI:** 10.1371/journal.pone.0097103

**Published:** 2014-05-12

**Authors:** Miso Park, Qing Sun, Fang Liu, Matthew P. DeLisa, Wilfred Chen

**Affiliations:** 1 Department of Chemical and Biomolecular Engineering, University of Delaware, Newark, Delaware, United States of America; 2 School of Chemical and Biomolecular Engineering, Cornell University, Ithaca, New York, United States of America; Universidad Nacional de La Plata., Argentina

## Abstract

The systematic organization of enzymes is a key feature for the efficient operation of cascade reactions in nature. Here, we demonstrate a facile method to create nanoscale enzyme cascades by using engineered bacterial outer membrane vesicles (OMVs) that are spheroid nanoparticles (roughly 50 nm in diameter) produced by Gram-negative bacteria during all phases of growth. By taking advantage of the fact that OMVs naturally contain proteins found in the outer cell membrane, we displayed a trivalent protein scaffold containing three divergent cohesin domains for the position-specific presentation of a three-enzyme cascade on OMVs through a truncated ice nucleation protein anchoring motif (INP). The positional assembly of three enzymes for cellulose hydrolysis was demonstrated. The enzyme-decorated OMVs provided synergistic cellulose hydrolysis resulting in 23-fold enhancement in glucose production than free enzymes.

## Introduction

In living organisms, many crucial cellular functions such as biosynthesis and cellular signaling [1] are controlled by multi-step enzymatic reactions that take place simultaneously with unsurpassed efficiency and specificity. A key characteristic of these highly efficient enzyme pathways is the cooperative and spatial organization of enzymes to ensure the sequential conversion of substrates [2]. This molecular-level organization of enzymes has a distinct enhancement effect on the overall efficiency by increasing the local enzyme and substrate concentrations, by channeling of intermediates between consecutive enzymes, and to avoid competition with other reactions present in the cell [3], [4]. One example is the Krebs TCA cycle, in which six of the eight sequential enzymes are clustered into a highly organized supramolecular complex [5]. Co-localization of enzymes can also be achieved by tethering enzymes to a linear protein scaffold such as the naturally occurring cellulosome structures. Formation of this highly ordered structure is mediated by the high-affinity protein-protein interaction (K_d_ <10^-9^ M) between the dockerin and cohesin domain, allowing the assembly of multiple cellulases in a spatially defined manner [6].

Attempts have been made to mimic this natural concept of enzyme assembly based on the use of either liposomes or polymersomes [7], [8]. These are spherical amphilphilic bilayer architectures of roughly 100 nm diameter that are synthetically assembled [9], [10]. Recently, a synthetic polymersome nanobioreactor has been created by positioning enzymes at three different locations, namely, in the lumen (glucose oxidase), in the bilayer membrane (lipase B), and on the surface (horseradish peroxidase). The enzyme-decorated polymersomes converted glucose acetate to hydrogen peroxide via a three-enzyme cascade reaction, illustrating the viability of advanced enzyme positioning using polymersomes [11]. Unfortunately, even this simple three-enzyme polymerosome assembly involves tedious and multiple-step syntheses and conjugations, making this approach impractical for large-scale applications.

Outer membrane vesicles (OMVs) are 20 to 200 nm proteoliposomes naturally derived from the surface of some gram-negative bacteria as part of their natural growth cycle [12]–[14]. These OMVs are compositionally similar to the bacterial outer membranes and contain lipopolysaccharides, outer membrane proteins, and phospholipids [14]. By taking advantage of the unique feature to embed outer membrane proteins into OMVs, a wide range of functional proteins such as green fluorescence protein (GFP) and β-lactamase (Bla) have been genetically tethered to the surface of hyper-vesiculating *Escherichia coli* and the corresponding OMVs using the virulence factor cytotoxin ClyA as the surface anchor [15]. Unlike complex enzyme assembly onto liposomes or polymerosomes, this results indicates the feasibility of designing OMVs as synthetic nanoreactors using only standard molecular biology techniques. Unfortunately, the ability to decorate OMVs with multiple functional protein moieties remains a major challenge as the competition for the same translocation pathway significantly reduces the expression of all target proteins.

**Figure 1:**
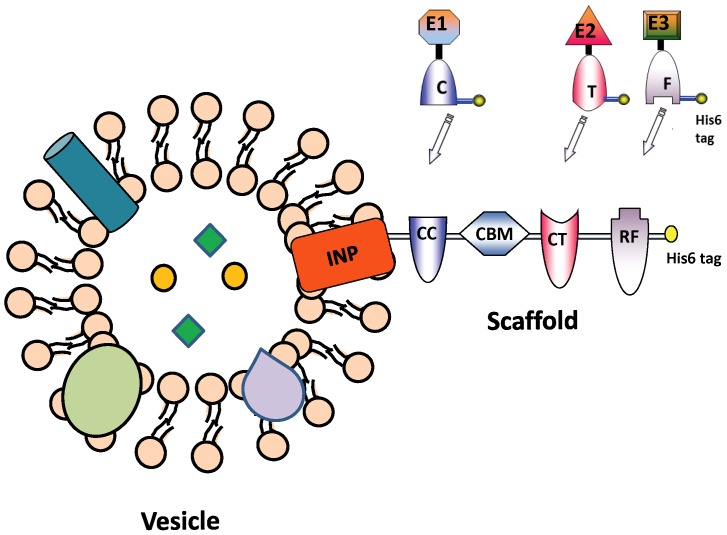
Functional assembly of multiple enzymes on engineered bacterial outer membrane vesicles (OMVs). A trivalent scaffold containing three orthogonal cohesin domains, DocC (from *C. cellulolyticum*), DocT (from *C. thermocellum*) and DocF (from *R. flavefaciens*), and one cellulose-binding module, is displayed onto OMVs using the ice nucleation protein (INP) anchor. The specific interaction between each cohesin-dockerin pair enables the sequential assembly of three dockerin-tagged cellulases (E1, E2 and E3) onto the OMVs at their corresponding position (C, T or F).

Many anaerobic bacteria have developed an elaborately structured multi-enzyme complex on the cell surface known as cellulosome for enhanced cellulose hydrolysis [16]. The main feature of this complex is a structural scaffold, which consists of repeating cohesin (Coh) domains that are docked individually with different cellulases tagged with a corresponding dockerin (Doc) domain. Inspired by this natural mechanism of enzyme assembly, artificial enzyme scaffolds have been designed for cellulose hydrolysis and methanol oxidation with up to 5-fold enhancement in efficiency [17], [18]. In this paper, we reasoned that a single protein scaffold based on Coh-Doc interactions can be used to sequentially assemble multiple enzymes onto the surface of OMVs (Fig. 1), resulting in nanoreactors capable of performing complex biocatalysis. Although the general framework can be applied to a wide range of multi-enzyme systems, the feasibility of the approach is illustrated using cellulose hydrolysis as a model system.

## Materials and Methods

### Construction of Plasmid and Expression of Protein Scaffold for Bacterial Surface Display

Plasmid pINP was constructed by ligating the PCR-amplified the truncated ice nucleation protein gene from pPNC20 [19] using primers INP-F (GGAATTCCATATGAATATCGACAAAGCGTTGGTA-CTGC) and INP-R (GCGCGAGCTCCTCGACCTCTATCCAGTCATCGTCC) into pET24a between NdeI and SacI sites. A trivalent scaffold, Scaf3 was amplified from pSctf [17] with primers scaf-F (GCGCGAGCTCCAACAGCAGCAATCCGGCGATTCTCTTAAAGTTACAGTAGGAACAGC) and scaf-R (TGCCGCTCGAGCTTAACAATGATAGCGCCATCAGTAAGAGTAACC) and inserted into pINP between SacI and XhoI, resulting in pINPScaf. The entire INP-scaf3 was amplified from pINPScaf with a forward primer IS-F (GCTCTAGAGAAGGAGATATACATATGAATATCGACAAAGCGTTG-GTACTGC) and a reverse primer IS-R (GCTCTAGATCAGTGGTGGTGGTGGTGGTGCTTAACAAT-GATAGCGCCATCAGTAAGAGTA) and the amplified fragment of INP-scaf3 was inserted into pVLT33 [19] with XbaI restriction site. Resulting plasmid, pINPscaf3 was confirmed by sequencing. Plasmid pINPscaf3 was transformed into the hyper-vesiculating mutant *E. coli* JC8031 [20]. Cultures of *E. coli* JC8031 harboring pINPscaf3 were grown in 100 ml of TB medium containing 50 µg/ml kanamycin at 37 °C. Protein expression was induced by the addition of 20 µM IPTG and grown at 30 °C when the OD600 reached ∼0.5.

### Immunofluorescence Microscopy

*E. coli* cells displaying Scaf3 were harvested by centrifugation, washed with phosphate buffered saline (PBS buffer, 10 mM, pH 7.4) and resuspended in PBS containing 0.1 % bovine serum albumin (BSA) and mouse anti-His immunoglobulin G (IgG, Applied Biological Materials). After 2 h incubation at room temperature with occasional mixing, cells were washed twice with the same buffer before resuspending in PBS with 0.1 % BSA and the goat anti-mouse IgG conjugate with Alexa488 (Invitrogen). Cells were washed twice after 1 h incubation, resuspended in PBS buffer, and examined by an Olympus BX51 fluorescent microscope.

### Isolation of Bacterial Outer Membrane Vesicles

Vesicles were collected from the cell-free supernatant as described [21]. In brief, cells were harvested at late-exponential phase and pelleted for 20 min at 4000 g and 4 °C. The cell-free supernatant was filtered through a 0.45 µm pore-size vacuum filter (Millipore). Vesicles were collected by centrifugation at 20,000 g and 40,000 g each for 30 min and 141,000 g for 3 h at 4 °C and resuspended in PBS or Tris buffer (50 mM, pH 7.5). For the density gradient separation, OMVs were loaded in different layers of Optiprep (Sigma) in Hepes (50 mM, pH 6.8) (35%, 30%, 25%, 20%, 15% and 10%) and centrifuged at 141,000 g for 3 h at 4 °C. Thirteen fractions of equal volume were sequentially taken out and analyzed.

### Transmission Electron Microscopy (TEM) and Dynamic Light Scattering (DLS)

Samples in PBS buffer were applied to 400-mesh carbon-coated copper grids (Electron Microscopy Sciences), stained with 2% uranyl acetate, air dried, and visualized on a Zeiss CEM 902 transmission electron microscope operating at voltage of 120 kV. Dynamic light scattering (DLS) for characterization of size of engineered vesicles was performed on a ZetaPALS (Brookhaven Instruments, Holtsville, NY). Collected vesicles were suspended in PBS buffer, and 1 ml of the vesicle suspension was transferred to a square cuvette for DLS measurement. DLS evaluates the time-dependent fluctuations of spherical particles in a light scattering intensity caused by the Brownian motion and a hydrodynamic diameter is calculated via the Stokes-Einstein equation [22]. The diameter of vesicles was calculated using BTC particle sizing software (Brookhaven Instruments, Holtsville, NY), demonstrating an average particle size of about 50 nm.

### Enzyme Expression and Assembly on OMVs

Construction of dockerin-fused cellulases were previously described [17]. *E. coli* BL21 (DE3) cells harboring each plasmid were precultured overnight at 37 °C in LB medium with 100 µg/ml of ampicillin or 50 µg/ml kanamycin. Cells were inoculated again in the same medium with 1.5 % glycerol before adding 200 µM IPTG at an OD600 of 1. After 16 h, cells were harvested by centrifugation and lysed with a sonicator in Tris buffer containing 100 mM NaCl and 10 mM CaCl_2_. For binding, OMVs were incubated with different cell lysates for 2 h at room temperature in the same Tris buffer as above. After incubation, OMVs were washed and collected by centrifugation (141,000 g, 30 min, 4 °C) and resuspended in the same buffer for protein analysis and activity assay.

### Enzyme Activity Assay

Enzyme activity was determined as described [17]. Briefly, enzyme activities were determined using either carboxymethyl cellulose (CMC) for endoglucanase or phosphoric acid-swollen cellulose (PASC) (prepared from Avicel pH101) for exoglucanase as the substrate. For β-glucosidase, cellobiose or 4-Methylumbelliferyl β-D-glucopyranoside were used. Reducing sugars were determined with DNS reagents (10 g/l dinitrosalicylic acid, 10 g/l sodium hydroxide, 2 g/l phenol, 0.5 g/l sodium sulfite). Samples mixed with DNS reagents were incubated at 95 °C for 10 min, and the absorbance was measured at 575 nm with a microplate reader (Synergy H4, BioTek). Glucose concentrations were determined with a glucose HK assay kit (Teco Diagnostics).

## Results and Discussion

### Expression and Cell Surface Display of Protein Scaffold (Scaf3)

**Figure 2:**
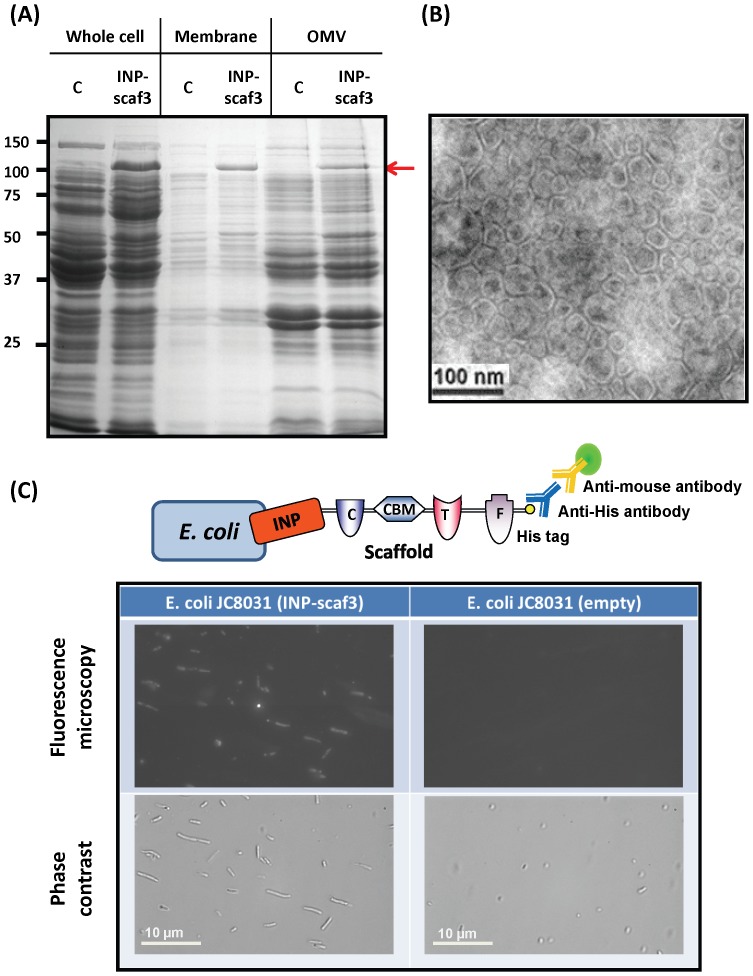
Expression and localization of INP-Scaf3. (A) SDS-PAGE analysis of whole cell, membrane and OMV fractions of *E. coli* JC8031 with (INP-scaf3) or without (C) INP-Scaf3 expression. The INP-Scaf3 band is indicated by an arrow (107 kDa). (B) Electron micrograph of engineered OMVs expressing INP-scaf3. The scale bar represents 100 nm. (C) Surface localization of INP-Scaf3. Cells expressing INP-Scaf3 were incubated with mouse monoclonal anti-his6 antibody and Alexa488-conjugated anti-mouse IgG. The scale bar represents 10 µm.

To display multiple functional proteins on the OMVs, a trivalent scaffold (Scaf3) comprised of three orthogonal cohesin domains from *Clostridium cellulolyticum* (C), *Clostridium thermocellum* (T) and *Ruminococcus flavefaciens* (F) and an internal cellulose-binding module (CBM) from our previous work was adopted [17]. Although Scaf3 have been successfully displayed onto the surface of *Saccharomyces cerevisiae* [17], it is unclear whether this large scaffold (over 100 kDa) can be functionally displayed on the surface of *E. coli*. To investigate this feasibility, the truncated ice nucleation protein anchor (INP), which has been used as an surface anchor to display proteins up to 119 kDa in *E. coli* [19], was used to target Scaf3 to the surface of the OMV-hyperproducing strain JC8031 (Fig. 1) [20]. This particular strain was chosen because the *E. coli* strain BL21, which was used to express the different cellulases, produced virtually no OMV under the same growth condition. Successful production of INP-scaf3 was demonstrated by SDS-PAGE analysis (Fig. 2). A new protein band (107 kDa) corresponding to the full-length INP-scaf3 was detected in both whole-cell and membrane fractions comparing to cells harboring only the empty vector (Fig. 2A). The surface localization of Scaf3 was confirmed using immunofluorescence microscopy by probing the C-terminus his6 tag on the scaffold (Fig. 2C). Cells displaying Scaf3 were brightly fluorescent while no fluorescence was detected for the control cells harboring the empty plasmid. It is interesting to note that cells displaying Scaf3 were significantly elongated, likely the result of the very high-level expression of INP-scaf3 in the cell membrane.

### Incorporation and Surface Display of Functional Scaf3 on OMVs

After confirming the surface localization of Scaf3, we next investigated whether the displayed INP-scaf3 can be incorporated into the secreted OMVs. As OMVs are released into the culture medium during cell growth, they can be easily isolated from cell-free culture supernatants using sequential centrifugations with increasing speeds up to 141,000 g and analyzed on SDS-PAGE (Fig. 2A). Similar to other previous reports, both engineered and non-engineered OMVs contained many native membrane and periplasmic proteins. However, a high level of INP-scaf3 was only detected within the engineered OMVs (Fig. 2A), indicating that the INP anchor not only targeted Scaf3 to the cell surface but also to the OMVs. Electron microscopy (Fig. 2B) revealed the presence of fairly uniform sized vesicles with an average diameter of 46.1±7 nm (as determined by dynamic light scattering), which are identical to OMVs produced by cells carrying the empty vector (data not shown) [15]. The engineered OMVs exhibited the typical spherical lipid bilayer morphology, indicating incorporation of the large scaffold has virtually no effect on the nano-sized spherical shape of OMVs. To confirm that INP-scaf3 was associated only with intact vesicles but not lysed membrane fragments, the collected vesicles were separated by density gradient centrifugation (Fig. S1 in File S1). Since the lumen of OMVs contains periplasmic proteins [13], presence of INP-scaf3 in intact vesicles was illustrated by the co-migration with both periplasmic (DsbA) and outer membrane (OmpA) proteins in the later fractions, an observation consistent with other reported engineered OMVs [15]. Detection of OmpA and/or DsbA alone in the earlier fractions indicated the presence of a small quantity of lysed membrane fragments or intact vesicles without any INP-scaf3 in the preparation.

**Figure 3:**
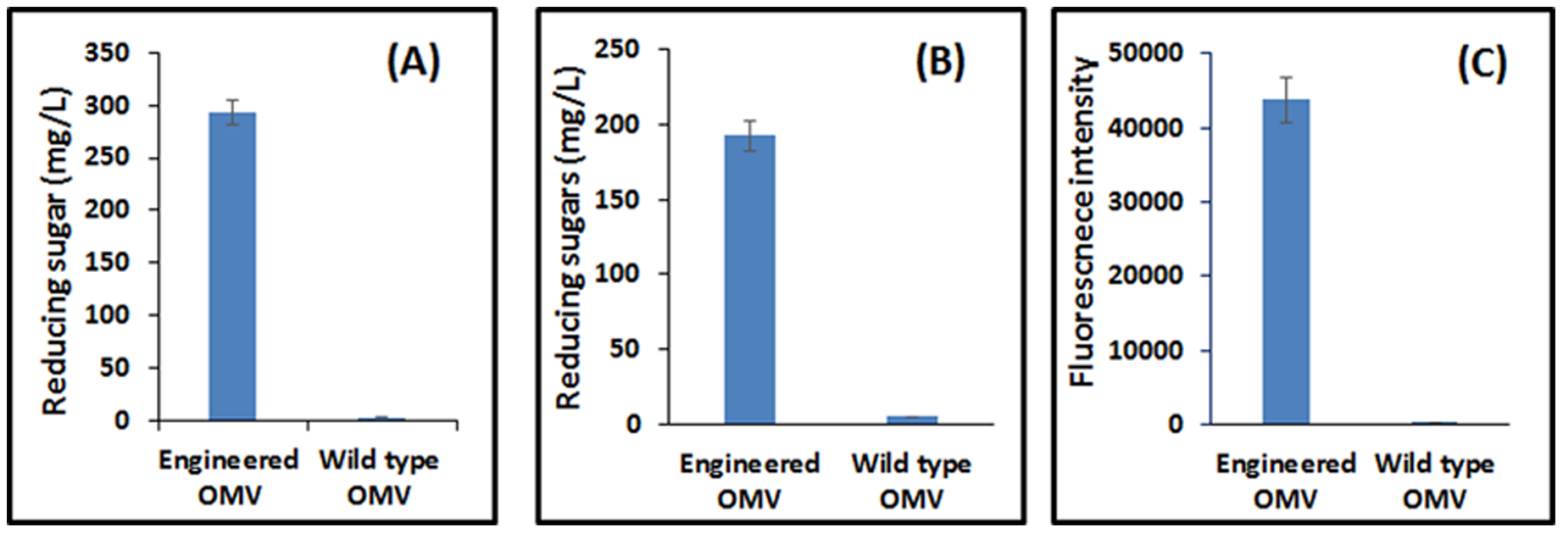
Binding of dockerin-tagged cellulases to engineered OMVs. Binding was verified by measuring the amount of reducing sugar released from cellulose hydrolysis after AT (A) or EC (B) binding. BF binding (C) was confirmed by measuring the fluroescence of 4-methylumbelliferone produced from the hydrolysis of 4-Methylumbelliferyl β-D-glucopyranoside. In all cases, wild type OMVs were used as a control.

**Figure 4:**
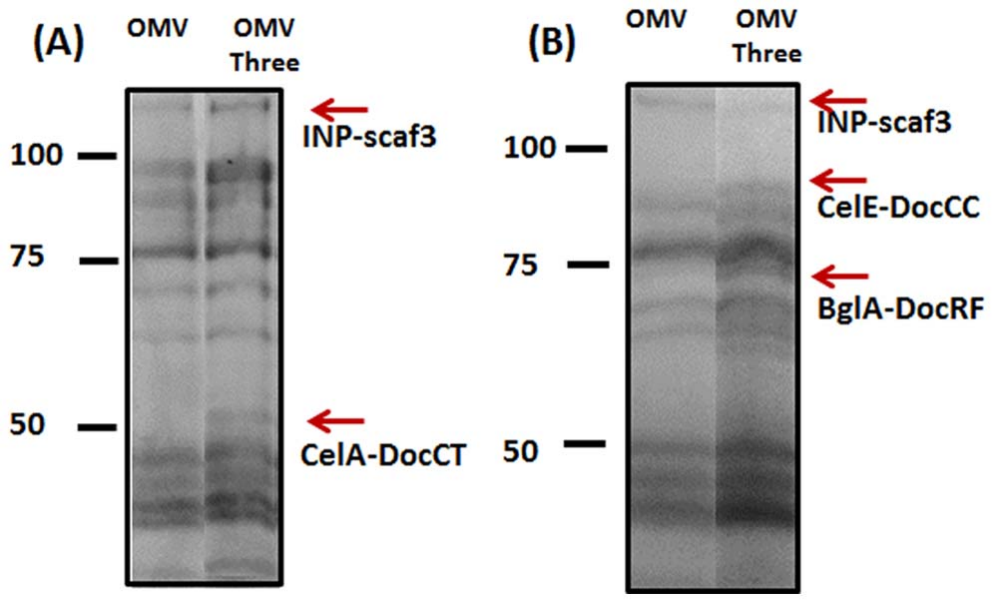
SDS-PAGE analysis of enzyme-assembled OMVs. For better resolution, binding of AT (A) or EC and BF (B) was confirmed using either 10% or 7% SDS-PAGE. Bands corresponding to either INP-scaf3 (107 kDa), AT (53 kDa), EC (90 kDa) or BF (70 kDa) are shown.

To probe the surface-accessibility and functionality of Scaf3, three different dockerin-tagged cellulases, an endoglucanase fused with DocT (AT), an exoglucanase fused with DocC (EC) and a β-glucosidase fused DocF (BF), were used. Each cellulase was individually assembled onto the OMVs. After removing unbound proteins, OMVs were washed and recovered by centrifugation. Enzyme docking onto Scaf3-decorated OMVs was verified by detecting enzyme activities for each cellulase (Fig. 3). In contrast, only background activity was detected for the native OMVs after the same incubation (Fig. 3 and Fig. S2 in File S1). This result confirms that the association between cellulases and OMVs was based solely on the specific Coh/Doc interaction and non-specific interactions between OMVs and enzymes are negligible. More importantly, only a single additional protein band corresponding to either AT, EC or BF was detected from the recovered OMVs (Fig. 4), while no new band was detected with the native OMVs (Fig. S2 in File S1). This result again demonstrates the specific recruitment of each enzyme onto the engineered OMVs even in a complex protein mixture.

### Multi-Enzyme Assembly on OMVs

The concept of sequential assembly of multiple enzymes onto OMVs was further demonstrated by assembling all three cellulases. Enzyme binding curves were first constructed with increasing amounts of cell lysates to determine the amount of cellulase required to saturate the corresponding cohesin domain on Scaf3 (Fig. S3 in File S1). In all cases, the OMV-associated activity continued to increase until reaching a plateau when all binding sites were occupied [18]; these binding conditions were used to assemble different multi-enzyme cellulosome structures onto the OMVs. Since a key feature of cellulosome is the ability to enhance cellulose hydrolysis over that of non-complex enzymes, the ability of the resulting OMVs carrying either AT, AT+EC, or all three enzymes to provide synergistic cellulose hydrolysis was assessed by comparing hydrolysis of phosphoric acid-swollen cellulose (PASC) to that of free enzymes.

**Figure 5:**
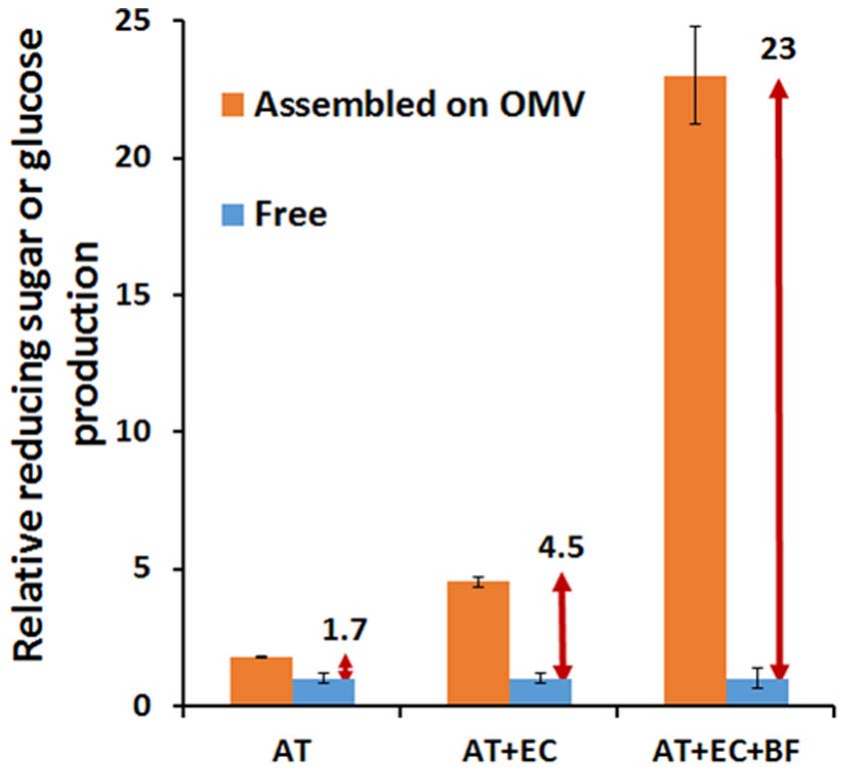
Production of reducing sugars (AT and AT+EC) or glucose (AT+BC+RF) from PASC by enzyme-assembled OMVs or the same amount of free enzymes.

Since the functionality of AT and EC is to cleave cellulose primarily into shorter chain oligomers with limited glucose liberation, we used reducing sugar production to assess the hydrolysis efficiency for OMVs carrying AT and EC. The amount of reducing sugar produced from OMVs carrying only AT was 1.7 times higher than the same amount of free AT (Fig. 5). This increase is due to the improved substrate accessibility and is consistent with the enhancement observed when a similar AT-based cellulosome was displayed on the yeast surface [17]. In contrast, when both AT and EC were co-assembled on the displayed scaffolds, the 4.5-fold enhancement in reducing sugar production over free enzymes is almost twice as that observed when the same two-enzyme cellulosome was displayed on the yeast surface (Fig. 5). This higher level of synergy indicates the importance of nanoscale structure in improving enzyme proximity and the cooperative action between adjacent enzymes. This benefit is more clearly demonstrated by the more than 23-fold higher level of glucose produced by OMVs carrying all three enzymes as compared to only 2.5-fold when the same trivalent cellulosome structure was displayed on the yeast surface (Fig. 5 and Fig. S4 in File S1). This substantially higher level of enzyme synergy again highlights the enhanced cooperative action between initial cellulose hydrolysis steps by AT and EC and the final conversion of cellobiose into glucose by BF using the nanoscale OMV structure because of the much higher enzyme to volume ratio.

## Conclusions

In summary, a synthetic biology strategy was used to generate nanoscale biological vesicles (OMVs) with multiple enzymatic activities. A trivalent protein scaffold was genetically tethered onto the OMVs to enable the positional specific recruitment of three different cellulases. The assembled enzyme complex not only retained full activity but also hydrolyzed cellulose 23-fold faster than non-complexed enzymes. Our synthetic biology approach excludes the use of any chemical conjugation and can provide a simple platform for generating nanobiocatalysts for highly efficient multi-enzyme reactions. The flexibility in designing scaffolds using a wide range of specific binding domains will permit the virtually unlimited number of functional proteins to be displayed on the OMVs.

## Supporting Information

File S1 **Supporting Figures.** Figure S1, Density-gradient fractionation of vesicles. Western blot analysis of different fractions using an anti-His6, anti-OmpA or anti-DsbA serum, respectively. Figure S2, Binding of CelA-DocCT onto wild-type and engineered OMVs. (A) SDS-PAGE analysis of OMVs before and after enzyme binding. (B) CelA activity of the resulting OMVs after binding. Figure S3, Hydrolysis of cellulose by single enzyme binding. Cellulose hydrolysis from the binding of increasing amount of CelA-DocT (CMC) (A), CelE-DocC (PASC) (B) and BglA-DocF (cellobiose) (C). Figure S4, SDS-PAGE analysis of OMVs loaded with all three enzymes. For better resolutions, binding of (A) AT or (B) EC and BF was confirmed using either 10% or 7% SDS-PAGE. Bands corresponding to either INP-scaf3 (107 kDa), AT (53 kDa), EC (90 kDa) or BF (70 kDa) are shown.
(DOCX)
