# Supplementary material for: Positional Assembly of Enzymes on Bacterial Outer Membrane Vesicles for Cascade Reactions
Source: PLoS One. 2014 May 12;9(5):e97103. doi: 10.1371/journal.pone.0097103 (PMC4018249; doi:10.1371/journal.pone.0097103)
Supplement: File S1 — Supporting Figures. Figure S1, Density-gradient fractionation of vesicles. Western blot analysis of different fractions using an anti-His6, anti-OmpA or anti-DsbA serum, respectively. Figure S2, Binding of CelA-DocCT onto wild-type and engineered OMVs. (A) SDS-PAGE analysis of OMVs before and after enzyme binding. (B) CelA activity of the resulting OMVs after binding. Figure S3, Hydrolysis of cellulose by single enzyme binding. Cellulose hydrolysis from the binding of increasing amount of CelA-DocT (CMC) (A), CelE-DocC (PASC) (B) and BglA-DocF (cellobiose) (C). Figure S4, SDS-PAGE analysis of OMVs loaded with all three enzymes. For better resolutions, binding of (A) AT or (B) EC and BF was confirmed using either 10% or 7% SDS-PAGE. Bands corresponding to either INP-scaf3 (107 kDa), AT (53 kDa), EC (90 kDa) or BF (70 kDa) are shown. (DOCX) [file pone.0097103.s001.docx]

**Supplementary Information**

Positional assembly of enzymes on bacterial outer membrane vesicles for cascade reactions

Miso Park,^a^ Qing Sun,^a^ Fang Liu,^a^ Matthew P. DeLisa^b^ and Wilfred Chen*^a^

*^a^* Department of Chemical and Biomolecular Engineering, University of Delaware, Newark, DE 19716

*^b^* School of Chemical and Biomolecular Engineering, Cornell University, Ithaca, NY 14853, USA


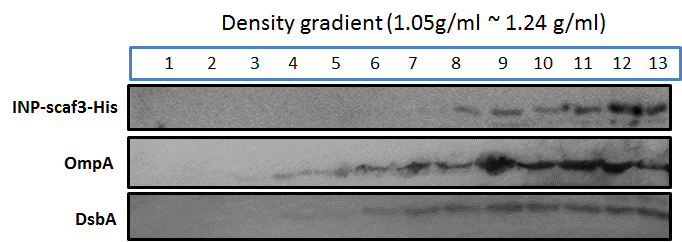
**Figures**

**Fig. S1.** Density-gradient fractionation of vesicles. Western blot analysis of different fractions using an anti-His6, anti-OmpA or anti-DsbA serum, respectively.

**B**

**A**

**Fig. S2.** Binding of CelA-DocCT onto wild-type and engineered OMVs. (A) SDS-PAGE analysis of OMVs before and after enzyme binding. (B) CelA activity of the resulting OMVs after binding.


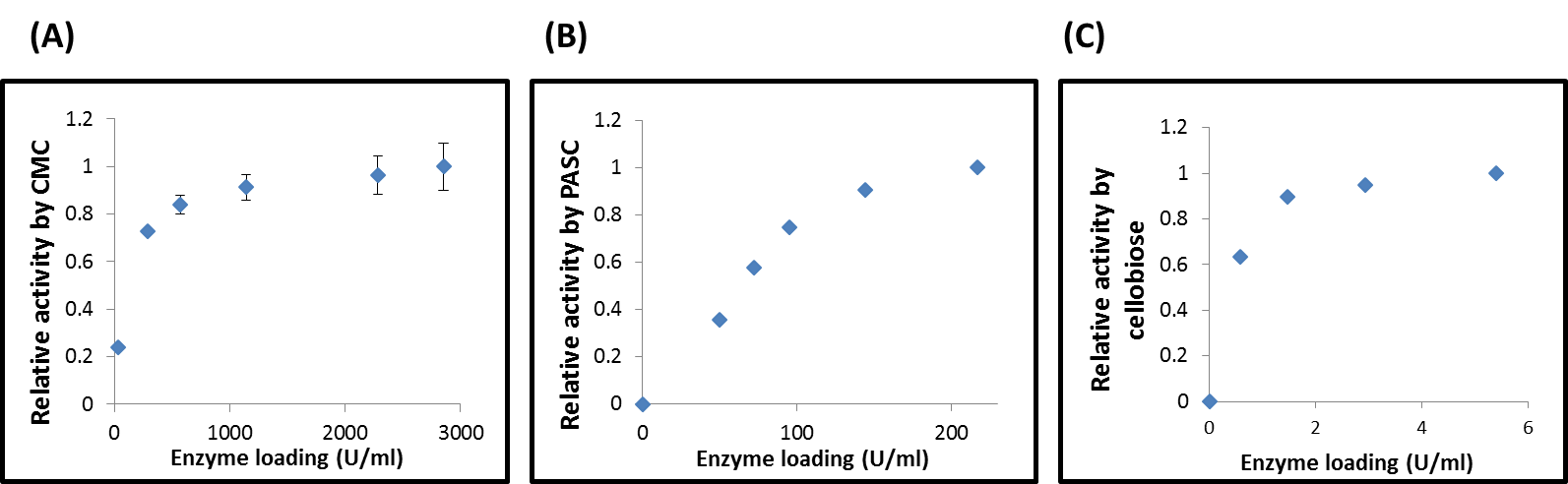


**Figure S3.** Hydrolysis of cellulose by single enzyme binding. Cellulose hydrolysis from the binding of increasing amount of CelA-DocT (CMC) (A), CelE-DocC (PASC) (B) and BglA-DocF (cellobiose) (C).


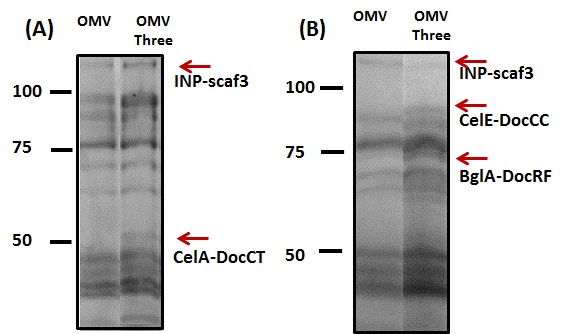


**Figure S4.** SDS-PAGE analysis of OMVs loaded with all three enzymes. For better resolutions, binding of (A) AT or (B) EC and BF was confirmed using either 10% or 7% SDS-PAGE. Bands corresponding to either INP-scaf3 (107kDa), AT (53kDa), EC (90kDa) or BF (70kDa) are shown.

**References**

1. W. Bae, A. Mulchandani, W. Chen, *J. Inorg. Biochem.* 2002, **88,** 223

2. S.-L. Tsai, J. Oh, S. Singh, R. Chen, W. Chen, *Appl. Environ. Microbiol.* 2009, **75**, 6087A

3 Bernadac, M. Gavioli, J. C. Lazzaroni, S. Raina and R. Lloubes, *J Bacteriol*, 1998, **180**, 4872

4 D. J. Chen, N. Osterrieder, S. M. Metzger, E. Buckles, A. M. Doody, M. P. DeLisa and D. Putnam, *P Natl Acad Sci USA*, 2010, **107**, 3099

5 R. C. Murdock, L. Braydich-Stolle, A. M. Schrand, J. J. Schlager, S. M. Hussain, *Toxicol. Sci.* 2008, **101**, 239
